# Job satisfaction criteria to improve general practitioner recruitment: a Delphi consensus

**DOI:** 10.1093/fampra/cmac140

**Published:** 2022-12-06

**Authors:** Bernard Le Floch, Hilde Bastiaens, Jean-Yves Le Reste, Patrice Nabbe, Perrine Le Floch, Mael Cam, Tristan Montier, Lieve Peremans

**Affiliations:** Department of Family Practice, University of Western Brittany, Brest, France; Department of Primary and Interdisciplinary Care, Faculty of Medicine and Health Sciences, University of Antwerp, Antwerp, Belgium; Department of Family Practice, University of Western Brittany, Brest, France; Department of Family Practice, University of Western Brittany, Brest, France; Department of Family Practice, University of Western Brittany, Brest, France; Department of Family Practice, University of Western Brittany, Brest, France; Department of Family Practice, University of Western Brittany, Brest, France; INSERM 1078 unit, SFR 148 ScInBioS unit, European University of Brittany, Faculty of Medicine and Health Sciences, Brest, France; Department of Primary and Interdisciplinary Care, Faculty of Medicine and Health Sciences, University of Antwerp, Antwerp, Belgium; Department of Nursing and Midwifery, Faculty of Medicine and Health Sciences, University of Antwerp, Antwerp, Belgium; Mental Health and Wellbeing Research Group, University of Brussels, Brussels, Belgium

**Keywords:** career choice, Delphi technique, family practice, general practitioner, job satisfaction

## Abstract

**Background:**

The clinical general practitioner (GP) workforce is decreasing. Many studies have analysed the negative aspects of the profession but, few examine the positive aspects and job satisfaction. A European collaborative group including 8 participating countries recently conducted a qualitative study to analyse the positive factors and found 31 job satisfaction factors.

**Objectives:**

To determine which of these 31 factors are important and applicable to future policies to improve family medicine attractiveness, recruitment, and retention in France.

**Method:**

The Delphi consensus method was chosen. Two Delphi rounds were conducted in March–April 2017 and retained satisfaction factors with at least 70% of scores ≥7. The Nominal Group Technique (NGT) was used to rank these retained factors. Participants assigned 5 points to the factor they considered most important, 3 points to the second, and 1 point to the third. Factors receiving at least 5% (10 points) of the total points (198 points) were included in the final list. The expert panel included GPs and non-GPs.

**Results:**

Twenty-nine experts began the procedure and 22 completed it. Thirty factors were retained after the 2 Delphi rounds. The NGT resulted in 8 factors: (i) Engage in family medicine to take care of the patients; (ii) Care coordination, patient advocacy; (iii) Flexibility in work; (iv) Trying to be a person-centred doctor; (v) Involvement in healthcare organization; (vi) Benefiting from a well-managed practice; (vii) Being a teacher, a trainer; (viii) Efficient professional collaboration.

**Conclusion:**

These 8 job satisfaction factors are important to consider and apply to future policy development.

**Key messages**

- Considering job satisfaction factors could improve GP retention and recruitment.
- Job satisfaction factors are important and applicable.
- Experts felt improving training in family medicine was important.
- As was developing care coordination, organization, and teaching.
- A patient-centred approach and flexibility in work are also crucial.
- As are well-managed practices and efficient professional collaboration.

## Background

The family medicine specialty has a specific, important, and complex role in the healthcare system to ensure high-quality primary care for the general population.^1,2^ General practitioners (GPs) provide family medicine, which includes prevention, screening, diagnosis, treatment, patient follow-up, and referral within the healthcare system.^3^ Strengthening family medicine supply improves population health and equal access to care while optimizing healthcare spending.^3,4^

For most countries, GPs are the first point of patient contact with the healthcare system.^4,5^ However, healthcare systems within Europe and the Organisation for Economic Co-operation and Development (OECD) countries are currently experiencing significant GP shortages affecting access to primary care. This is due to the declining physician population and student disinterest in this speciality.^1,6^ In France, the National Council of the Order of Doctors published the atlas of medical demography, which revealed a decreasing number of regularly practicing GPs and poor generational renewal.^7,8^ Many studies have stressed the negative aspects of the specialty to explain these GP shortages.^9^ However, positive aspects and job satisfaction have been poorly studied.^10^

Job satisfaction is particularly important since GPs with higher job satisfaction are less likely to experience burnout and job-related stress, and will stay in their job for longer.^11^ Furthermore, they are more likely to find their work interesting and be involved in work-related decisions.^12^

To conduct further research into job satisfaction, a European collaborative research group was created within the European General Practice Research Network (EGPRN).^13^ The group was co-directed by Brest and Antwerp Universities and included Germany, Belgium, Bulgaria, Finland, France, Israel, Poland, and Slovenia. They conducted a descriptive, qualitative study to highlight the positive factors determining job attractiveness and retention in family medicine. Overall, 183 GPs were interviewed in the 8 different countries. Analysis identified 31 job satisfaction factors linked to the profession.^14^

To successfully improve GP retention and recruitment, mobilizing stakeholders is necessary but not sufficient. To change things in reality, health policy makers must apply these positive factors.^15^ However, it is important to bridge the gap between research and policy makers and translate these results into usable findings.

This study aims to determine expert consensus regarding which of these 31 GP job satisfaction factors are relevant in terms of importance and applicability for future policies to improve attractiveness, recruitment, and retention in family medicine in France.

## Methods

### Delphi consensus and Nominal Group Technique

For this study, a Delphi consensus method was used followed by the Nominal Group Technique (NGT) method to rank the factors.^16^

The Delphi consensus is defined as a general agreement amongst members of an expert group, which does not have to be unanimous.^17–21^ An expert is any person with a good knowledge of the subject and a legitimacy to express a representative opinion of the stakeholder group to which they belong.^22^

The 4 characteristics which make a Delphi consensus stand apart from other decision-making processes are expert input, iteration with controlled feedback, statistical group response, and anonymity. The consensus is based on quantified analysis of the answers, supplemented with qualitative information from participant justifications and comments. Before each round, the experts receive the results about statements where no consensus was reached in the previous round and can modify their answers.^20^ Participant anonymity is required to avoid dominance, authority, or affiliation phenomena.

The NGT is a well-known technique used successfully in several previous studies.^17,23–25^

### Preliminary work

The French research group created for this study consisted of 4 GPs from the Brest Faculty of Medicine who were experienced in qualitative research (BLF, JYLR, MC, and PLF). This research group performed the preliminary work which involved translating the 31 job satisfaction factors from the recently conducted qualitative study^26^ from English into French and writing an explanatory description for each factor. The factors and explanations were emailed to the panel of experts (Table 1).

**Table 1.** The 31 satisfaction factors resulting from the European collaborative research group qualitative study (2010) to be evaluated by the expert panel in the Delphi rounds (2017).

The 31 satisfaction factors to be evaluated in the Delphi rounds.		
Themes	Satisfaction factors	
A. GP as a person	1	Love job
	2	Taking care of yourself as a person
	3	Engage in family medicine to take care of patients
	4	Ability to cope
B. Special skills/competencies needed in practice	5	A highly intellectual profession
	6	Care coordination, patient advocacy
	7	Efficient communication skills
	8	Broad scope of activities
C. Freedom in work organization	9	Efficient professional collaboration
	10	Freedom of choice in workplace
	11	Involvement in the healthcare organization
	12	Benefiting from a well-managed practice
	13	Flexibility in work
D. Doctor–patient relationships	14	Patients’ gratitude
	15	Longitudinal care
	16	Trying to be a person-centred doctor
	17	Successful negotiations with patients
	18	Rich human relationships with patients
	19	Mutual trust and respect in doctor–patient relationships
	20	Being a doctor for the whole family
E. Teaching and learning	21	Continuing Professional Development
	22	Being a teacher, a trainer
	23	Positive role modelling of senior GPs
	24	Make practice attractive for young GPs
	25	Recognition of General Practice as a speciality
	26	Mutual benefits of GPs and trainees
F. Supportive factors for work–life balance	27	Positive experiences at the beginning of career
	28	A harmonious private life
	29	Job security
	30	A fair earnings/workload balance
	31	General Practice as a respected profession

The questionnaire used to assess the satisfaction factors was created and uploaded using Google Forms. It was initially emailed to 10 volunteers to test its feasibility and ensure it was understandable.

### Panel of experts

Panel members recruited for this study included people with relevant expertise or experience in family medicine and/or healthcare systems. They were recruited from 8 groups including GP trainee unions, GP unions, family medicine university departments, health system administrators, local elected representatives, Ministry of Health officials, specialized journalists, and patient organizations to ensure multidisciplinary representation from clinical, research, and patient member groups.

The research group contacted participants individually, by email or telephone, and were recruited through direct contact and a snowball effect. The aim was to contact 30 experts since at least 15 experts were needed for the Delphi rounds and 10 for the NGT. During this first contact, the study objective and method were explained. There was no relationship between participants and researchers prior to the study. The study protocol and a consent form were sent individually to each expert recruited in the leadup to the first phase of the study.

### Study procedure

#### Delphi procedure

The first Delphi round was conducted in March–April 2017. Participants had 3 weeks to respond. They had no contact with each other, and the Delphi procedure was anonymous. For each satisfaction factor, participants were asked to evaluate its relevance in terms of importance and applicability to support GP recruitment. They were asked to score each factor using a Likert scale^21^ ranging from 1 (not at all relevant) to 9 (very relevant). For any answer scoring below 7, they were asked to propose a reformulated explanatory description to improve acceptability or provide a comment explaining their low score. Factors with at least 70% of responses scoring between 1 and 3 were permanently removed from the study. Factors with at least 70% of responses scoring above or equal to 7 (consensus) were definitively kept for the next study phase. After the first round the research group met to discuss the results. The explanatory description for each factor not reaching consensus was reformulated following participant comments to make them more acceptable.

In the second Delphi round, only satisfaction factors which scored between 4 and 7 were re-evaluated but, no comments were asked for regardless of the score. The second round started on 14 April 2017, and finished on 29 April 2017. At the end of this second round, only factors with at least 70% of responses scoring 7 or more were selected for the NGT and were added to the factors that had already reached consensus in the first round.

#### NGT procedure

The third and final phases of the study, the NGT, were conducted by open email. The objective was to rank the satisfaction factors which reached consensus from the 2 Delphi rounds. Each participant was asked to rank the 3 statements they considered the most important and applicable to support GP recruitment using 3 scores: 5 points for the first, 3 points for the second, and 1 point for the third. The satisfaction factors were sent by email on 3 May 2017, and the results were collected on 24 May 2017.

Participants were invited to explain their choice and could comment on each other’s choices as many times as desired. They could modify their ranking until the end of the NGT process. Rankings were updated daily to encourage active debate, which is the aim of the NGT. Furthermore, the research group actively encouraged debate, taking care not to influence.

A hierarchical list of all satisfaction factors was established based on the total scores. Factors receiving at least 5% (10 points) of the total points distributed (198 points) were included in the final list. All participants received this final list for their information.

## Results

**Fig. 1.**
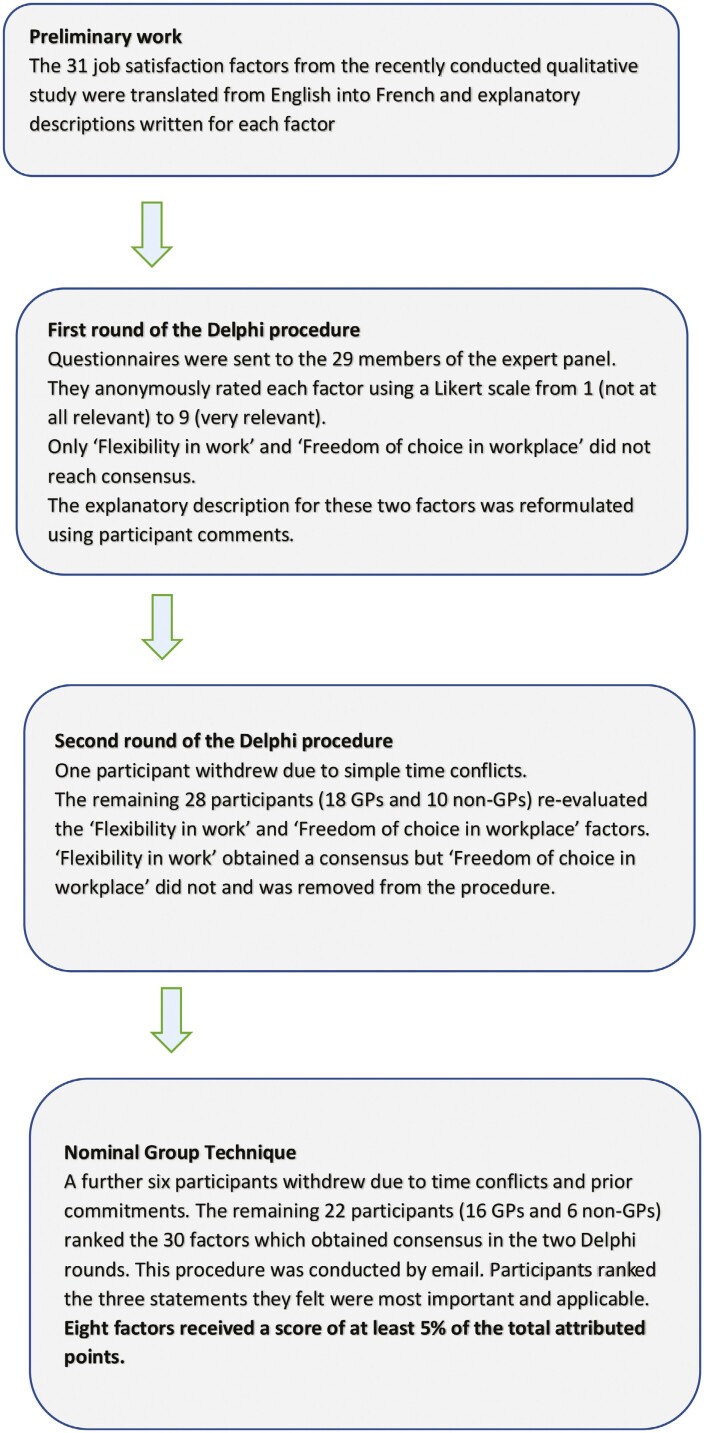
Flowchart to show the study process including the 2 Delphi rounds and the NGT (2017).

Expert recruitment was conducted from 24 February to 22 March 2017. Forty-one experts were contacted, of which 33 responded and 32 agreed to participate (Table 2). Twenty-nine participants responded in the first Delphi round, 28 in the second round, and 22 participated in the NGT (Fig. 1). All expert categories were represented in this final sample.

**Table 2.** The 29 experts who participated in the Delphi consensus rounds to determine the importance and acceptability of the 31 job satisfaction factors (2017).

Number	Group	Function	Gender Male (M)/Female (F)	GP or non-GP
1	Health ministry	Directorate General of Health	M	Non-GP
2	Elected Local	Local Elected in a rural community	M	Non-GP
3	Elected Local	Mayor of a rural town	M	Non-GP
4	Elected Local	Former Senator and mayor of a rural town	M	Non-GP
5	Elected Local	Senator and mayor of a rural town	M	Non-GP
6	Elected Local	City Council member	M	GP
7	Medical student union	Medical GP student union	F	GP
8	Medical student union	Medical GP student union	F	GP
9	Medical student union	Medical GP student union	F	GP
10	Medical student union	Former National President of GP student union	M	GP
11	Medical student union	National Manager of GP student union	F	GP
12	GP union	National General Medical Council	M	GP
13	GP union	Regional Union of Health Professions	M	GP
14	GP union	National Manager of French College of GPs	M	GP
15	GP union	President of a Regional Union of Health Professions	M	GP
16	GP union	National General Medical Council	F	GP
17	Academic GPs	Professor Family medicine St Etienne	M	GP
18	Academic GPs	National Manager of National College of GP teacher (French WONCA)	M	GP
19	Academic GPs	National College of GP teacher (French WONCA)	M	GP
20	Academic GPs	Director General Medicine Department in Family medicine. University Professor	M	GP
21	Academic GPs	Inter-Regional Coordinator of the Diploma of Studies in Family medicine. University Professor	M	GP
22	Academic GPs	Former Inter-Regional Coordinator of the Diploma of Studies in Family medicine. University Professor	M	GP
23	Academic GPs	Professor Family Medicine, Paris	F	GP
24	Health system	Regional Health Agency, Brittany, France	F	Non-GP
25	Health system	Manager of Local Health Insurance	M	Non-GP
26	Health system	Regional Health Agency in France	M	Non-GP
27	Health system	Medical control doctor Agricultural health insurance	M	Non-GP
28	Society	National television journalist. Specialist in health problems	F	Non-GP
29	Society	National President of a Patient Association	M	Non-GP

GP: Genral Practicer; WONCA: World Organization of National Colleges, Academies and Academic Associations of General Physician/Family Physicians.

### Results of the first Delphi round

After the first Delphi round, only 2 out of 31 satisfaction factors did not reach consensus: “Freedom of choice in workplace” and “Flexibility in work” (Table 3).

**Table 3.** Results of the first Delphi round assessing the 31 satisfaction factors.

Satisfaction factor	Number of scores ≥7	% of scores ≥7
Rich human relationships with patients	28	96.5
Mutual benefits of GPs and trainees	28	96.5
Ability to cope	27	93.1
Trying to be a person-centred doctor	27	93.1
Make practice attractive for young GPs	27	93.1
Involvement in the healthcare organization	27	93.1
Love job	26	89.6
Taking care of yourself as a person	26	89.6
Care coordination, patient advocacy	26	89.6
Efficient communication skills	26	89.6
Broad scope of activities	26	89.6
Mutual trust and respect in doctor–patient relationships	26	89.6
A fair earnings/workload balance	26	89.6
Engage in General Practice to take care of patients	25	86.2
A highly intellectual profession	25	86.2
Efficient professional collaboration	25	86.2
Benefiting from a well-managed practice	25	86.2
Successful negotiations with patients	25	86.2
Continuing Professional Development	25	86.2
Being a teacher, a trainer	24	82.7
Positive role modelling of senior GPs	24	82.7
Positive experiences at the beginning of career	24	82.7
Being a doctor for the whole family	23	79.3
Recognition of family medicine as a speciality	23	79.3
Family medicine as a respected profession	23	79.3
Longitudinal care	22	75.9
Job security	22	75.9
Patients’ gratitude	21	72.4
A harmonious private life	21	72.4
Freedom of choice in workplace	20	69.0
Flexibility in work	19	65.5

“Freedom of choice in workplace” received 20 scores (69%) higher than or equal to 7. The original explanation was: “*It is important that the GP can choose where they settle considering themselves, their family, environment, and the colleagues with whom they will collaborate. They choose their practice location according to their personal affinities with the community, their origins, or the opportunities*.” Reading the comments, “Freedom of choice in workplace” was complex and controversial. Several participants commented extensively on this factor, with several possible answers for each. Some wanted control to avoid additional GPs settling in areas with an overabundance of GP practices, whereas others ruled out settling in areas with too many GPs. Some experts suggested providing incentives for GPs to settle in specific areas as a potential solution. Experts also highlighted the social duty of the GP and their responsibility to organize healthcare. The commentary was rich, interesting, and constructive, which made it difficult to integrate all comments into a succinct explanation. However, the research group reformulated the explanation as follows: *Freedom of choice in workplace: It is important that the GP is not constrained in their choice of workplace, for them or their family. Choosing a workplace should be encouraged according to population needs, GP affinities with the population, and opportunities.*

For “Flexibility in work,” 19 scores (65.5%) were higher than or equal to 7. The original explanation was: “*GP activity covers all areas of medicine. Patients are varied. GPs must be given access to complementary university training: ultrasound, technical acts*.” Participant comments were varied. Several participants felt this factor was irrelevant because it is relative to and dependent on having available healthcare nearby. Some highlighted an already busy schedule, which sometimes compromises possibilities for additional training. Several experts felt that complementary training was not essential and, above all, depended on provision of care. The research group reformulated the explanation as follows: *Flexibility in work: GP activity covers different fields of medicine. Patients are varied. GPs should be given access to complementary university courses according to their activity, the needs of the population and the provision of care.*

### Results of the second Delphi round

Twenty-eight participants responded to the second Delphi round of which 18 were GPs and 10 non-GPs. One participant withdrew from the process. “Flexibility in work” obtained a consensus with 23 scores greater than or equal to 7 (82.1%). However, “Freedom of choice in workplace” did not reach consensus with only 19 scores higher than or equal to 7 (67.8%). However, it just reached consensus (70%) in the non-GP population.

### Nominal group results

In the NGT, there were 22 participants of which, 16 were GPs and 6 were non-GPs. Six participants withdrew from the process at this stage. At the end of the procedure, 8 factors received a score of at least 5% of the total points attributed (Table 4). These satisfaction factors are shown below including a summary of why the experts thought the statement could support the recruitment of young people into the profession.

**Table 4.** Results from the NGT to rank the most important satisfaction factors.

Ranking	Satisfaction factor	Score
1	Engage in general practice to take care of patients	29
2	Care coordination, patient advocacy	22
3	Flexibility in work	20
4	Trying to be a person-centred doctor	15
5	Involvement in the healthcare organization	14
6	Benefiting from a well-managed practice	14
7	Being a teacher, a trainer	13
8	Efficient professional collaboration	10

Eight factors received at least 5% of the total attributed points and were included on the final list (2017).

1. Engage in family medicine to take care of patients

Several experts put forward this factor as an essential element of the profession: “*it is obvious, but it feels better saying it*.” The notion of a “*quality relationship*” with the patient is reflected. Students who have “*interpersonal and patient-centred qualities*” will become a “*competent GP*.” One expert pointed out that “*the family medicine speciality best meets this motivation*.”

2. Care coordination, patient advocacy

The GP personalizes the care pathway for each patient and provides comprehensive, patient-orientated care. It is a “*specificity of the profession, which is stimulating on an intellectual, organisational and operational level*.”

3. Flexibility in work

Professional diversity appears to be a major factor in family medicine recruitment. The profession involves a wide field of activity, seen as “*a guarantee against the monotony of practice*.” This aspect of the profession makes it attractive to “*young people*,” and demonstrates a “*liberated*” profession, “*which can evolve through complementary training*.” This diversity is seen as “*an asset and a difficulty*,” which requires some preparation, training, and adaptability. This factor, which reached consensus after reformulation in the second Delphi round, appears a priority to improve recruitment in family medicine.

4. Trying to be a person-centred doctor

The “*global*” approach, centred on the patient, “*induces satisfaction in the human relationships that make up the richness of the profession*” and is the key to a “*useful practice for the community*.” It requires “*collaboration with other specialists and other health professionals*.”

5. Involvement in the healthcare organization

The participants stated that GPs have an active role in the care system, not a “*simple operator*” position.

6. Benefiting from a well-managed practice

It is “*the essential prerequisite for responsible and fulfilling practice*.” Some choose not to be subject to a hierarchical or institutional framework to feel “*free*.” This work organization “*allows GPs to practise with an adaptable schedule, and to find a work-life balance*.”

7. Being a teacher, a trainer

This factor demonstrates a certain unanimity. “*Students have a better understanding of the profession*.” “*Sharing knowledge and know-how*” and “*patient-centred strategies and techniques*” are “*a priority if we want to recruit motivated young physicians*.” This requires “*supervisor training*.” It is rewarding for trainers to see graduate students starting a career as a GP after discovering the profession through internships at their practice. One expert said: “*How can you decide on a specialty if you can’t discover it?*” Students also value the internships where the “*training is one of the most appreciated and best rated among the graduate placements*.” The family medicine internship enables aspects of the profession to be discovered which until then had not been covered by the hospital training. Being in contact with students “*leads to questions, training, and research in primary care, which is also an important factor for improving the speciality and providing an increased sense of competence*.”

8. Efficient professional collaboration

A fair work environment, and good relationships within the practice and with external specialist colleagues are essential for job retention. Building a good professional network is also important. This can begin with internships as a student in the potential area where the student wants to work. Communication between primary care and hospitals is important for organizing patient care pathways and for practice efficiency. For this, meetings and training between different professionals is beneficial.

In the GP population, “Taking care of yourself as a person” and “Rich human relationships with patients” received the most points.

## Discussion

### Main results

This is the first study in France to determine expert consensus regarding key job satisfaction factors for GPs. This is important for future policy development to improve family medicine attractiveness, recruitment, and retention in France.

The panel of experts was presented with 31 job satisfaction factors in family medicine from a recently conducted European qualitative study.^26^ They determined which factors were relevant to improve GP recruitment and retention. Each expert had to understand how the factors could improve the family medicine workforce in terms of economic, social, and political positioning.

Overall, the participants validated most satisfaction factors in family medicine as potential recruitment and retention factors. Experts felt that recruitment and retention would be more successful if family medicine was patient centred, and GPs had the tools and means to treat patients and take care of themselves. There was no initial consensus on “Flexibility in work” and “Freedom of choice in workplace” although the European qualitative study^14^ found these to be the most important factors for recruiting potential GPs.

### Strengths and limitations

The Delphi method and the NGT are well-recognized techniques for reaching consensus on the relevance of these satisfaction factors in terms of importance and applicability.^16^ Data from the previous studies and information from the experts could be integrated.^26^ The questionnaires were developed to limit agreement and opposition biases.^27^ Consensus was achieved if the minimum proportion of agreement reached 70% which is consistent with data in the literature.^21,23,24^

The panel of experts included multidisciplinary representation from clinical, research, and patient member groups. This was important to ensure multiple perspectives were considered and contributed to determining the key job satisfaction factors for GPs, which could then be used to inform future policy development. Experts were motivated by the need to improve recruitment in family medicine and medical demography. Participation was very active, showing the experts’ interest in this subject. There was geographic and socio-professional variation within the expert panel, although about two thirds of the experts lived in the West of France.

The response rate was very good with 32 participants from the 40 people contacted. Group size was consistent with Delphi method literature^18,27^ with 29 experts participating in the first round and 28 in the second. The NGT response rate was also good with 22 participating experts which is an optimal and effective size according to the literature.^21^ A larger group may have caused confusion in the debate. Lost to follow-up (6 experts) could be explained by time conflicts with prior commitments and the NGT being carried out on an open mailing list, which could cause a loss of confidentiality. Email was used because it was impossible to organize face-to-face meetings with experts from several regions with busy schedules. This might be a limitation because there was less interaction than in a group setting.

The data for the study were collected several years ago. However, the results are unlikely to be different today.^28^

### Results interpretation

#### First and second Delphi rounds

In the first round, “Flexibility in work” did not receive consensus. GPs saw flexibility in work as increasing their workload and pressure when they already had a busy schedule. Therefore, the researchers reformulated the explanation to indicate that “Flexibility in work” meant using a variety of skills to overcome challenges faced when practising primary care.

“Freedom of choice in workplace” is a sensitive, complex, and controversial issue involving the GP as a person and their relationship with the community.^29,30^ According to the protocol, only factor explanatory descriptions were modifiable, not the factor title. However, despite a new explanatory description, a consensus was not reached during the second Delphi round.

This study cannot solve the debate about GPs having freedom to choose a workplace. Changes do need to be made as the situation cannot remain as it is. There is a wide range of suggested solutions including the freedom to set up incentives and excluding areas with high GP density.^31,32^ However, some respondents had concerns that GPs cared more about where they choose to work than the population problems, suggesting social responsibility was lacking. The solution will undoubtedly be complex, but it is important to find one soon.

#### Nominal group results

“Engage in family medicine to take care of patients” received the highest score in the NGT. All the comments agreed that patient care is the main goal. A holistic, patient-centred approach including psychological, social, and cultural factors is particularly important in family medicine.

“Care coordination, patient advocacy” received the second highest score. This is not surprising as GPs act as a point of contact and ensure the care pathway runs smoothly. Furthermore, one of the main GP competencies is primary care management with the ability to coordinate between primary and secondary care.^2^

It is remarkable that “Flexibility in work” appears in third position since consensus was difficult to obtain in the first Delphi round. However, this topic is one of the most frequently described themes for GPs^10,30,33,34^ and is an aspect of the profession which makes it more attractive. It is less surprising that “Trying to be a person-centred doctor” featured on the list as this is an essential part of family medicine^2^ and is a challenging and everyday part of practise.

Our study agrees with numerous studies that “Being a teacher, a trainer” is an important factor to attract young doctors to family medicine.^35–37^ Teaching, sharing knowledge and experience, and mentoring were found to be rewards of family practice in a Canadian qualitative study using the Delphi method.^20^

### The influence of the COVID-19 pandemic

The constraints related to the COVID-19 pandemic have probably impacted GP satisfaction and practise became more difficult. However, GPs continued to fulfil their duty. During the pandemic, the following factors became even more important: “Engage in family medicine to take care of the patients,” “Care coordination, patient advocacy,” and “Involvement in healthcare organization.” This enabled GPs to be involved in COVID care, diagnosis, vaccinations, and prevention advice. In fact, many of the results from a study conducted from March to September 2021 on the well-being of Australian GPs are similar to those of our study.^28^

### Implications for policy makers and research

Given the concern about the primary care workforce and “medical deserts,” participating experts agreed that the satisfaction factors found in family medicine represent priority areas to support GP recruitment and that Health Authorities could use these in their efforts to effectively improve the family medicine workforce in France.

This study was not appropriate to address the complex “Freedom of choice in workplace” problem meaning further research is needed.^32^

The protocol for this study could be used by other European countries to promote GP recruitment by developing a list of factors considered important and applicable to their country from the health decision-maker point of view.

## Conclusion

This study determined expert consensus regarding key job satisfaction factors for GPs in France. These included: (i) Engage in family medicine to take care of patients; (ii) Care coordination, patient advocacy; (iii) Flexibility in work; (iv) Trying to be a person-centred doctor; (v) Involvement in the healthcare organization; (vi) Benefiting from a well-managed practice; (vii) Being a teacher, a trainer; (viii) Efficient professional collaboration. These factors are important to consider and apply to future policy development to improve attractiveness, recruitment, and retention in family medicine in France.

## Supplementary Material

cmac140_suppl_Supplementary_Checklist

## Data Availability

All data and materials can be sent on request. Contact Département Universitaire de Médecine Générale, Faculté des Sciences de la Santé, 22, avenue Camille Desmoulins, 29200 Brest Cedex, France.
